# Successful treatment of anti-neutrophil cytoplasmic antibody-associated inflammatory pseudotumour with rituximab: A case report

**DOI:** 10.1097/MD.0000000000045044

**Published:** 2025-10-03

**Authors:** Lan Gao, Hua Zhao, Chaoyang Liang, Honghu Tang

**Affiliations:** a Department of Rheumatology and Immunology, The First People’s Hospital of Yibin, Yibin, China; b Department of Rheumatology and Immunology, West China Hospital Sichuan University, Chengdu, China; c Department of Rheumatology and Immunology, Jiange First People’s Hospital, Guangyuan, China.

**Keywords:** ANCA-associated vasculitis, ocular vasculitis, orbital inflammatory pseudotumor, rituximab, sclerosing OIP, steroid dependence

## Abstract

**Rationale:**

Anti-neutrophil cytoplasmic antibody (ANCA)-associated vasculitis (AAV) is a rare autoimmune condition that can involve various organ systems. Ocular involvement, though uncommon, may present as the initial or even sole manifestation. Orbital inflammatory pseudotumor, particularly its sclerosing subtype, can pose significant diagnostic and therapeutic challenges due to its steroid dependence and potential for recurrence.

**Patient concerns:**

A 72-year-old Chinese man presented with a 6-month history of recurrent headaches. He later developed bilateral exophthalmos and progressive vision loss despite initial treatment. His past medical history included type 2 diabetes mellitus.

**Diagnoses:**

The patient initially tested positive for both p-ANCA and c-ANCA, with markedly elevated anti-myeloperoxidase and proteinase 3 antibody levels. Imaging revealed bilateral orbital masses consistent with orbital inflammatory pseudotumor, and biopsy confirmed sclerosing orbital inflammatory pseudotumor. Despite serological remission, clinical symptoms worsened during steroid tapering, suggesting steroid dependence.

**Interventions:**

The patient was initially treated with high-dose corticosteroids and oral cyclophosphamide. Although symptoms improved transiently, relapse occurred upon tapering steroids. Rituximab (RTX; 500 mg weekly for 4 weeks) was then administered as second-line therapy.

**Outcome:**

The patient experienced significant improvement following RTX therapy, which allowed successful steroid tapering. No further relapse occurred during short-term follow-up.

**Lessons:**

This case illustrates the limitations of cyclophosphamide-based regimens in managing steroid-dependent ANCA-associated orbital disease. RTX may be a more effective alternative in relapsing or refractory cases, particularly when ocular symptoms persist despite ANCA seronegativity. Early recognition and individualized immunosuppressive therapy are essential for preserving vision and improving outcomes in ocular ANCA-associated vasculitis.

## 1. Introduction

Anti-neutrophil cytoplasmic antibody (ANCA)-associated vasculitis (AAV) is a group of autoimmune diseases characterized by systemic or localized inflammation predominantly involving small- to medium-sized blood vessels. In autoimmune diseases, breakdown of immune tolerance leads to the production of pathogenic autoantibodies that target self-antigens and cause tissue injury. In systemic lupus erythematosus, a prototypical systemic autoimmune disease, diverse autoantibodies—such as anti-dsDNA, anti-Sm, and antiphospholipid antibodies—arise from aberrant B-cell activation and persistent antigenic stimulation, contributing to multi-organ involvement and cumulative organ damage, which is a major determinant of long-term prognosis.^[1]^ Similar immune dysregulation underlies antibody production in other autoimmune disorders, including AAV, in which ANCAs drive vascular inflammation and organ injury. Ocular involvement can occur in isolation or alongside systemic manifestations and may affect individuals of any age, including those without detectable systemic disease. ANCA-associated ocular disease is notably heterogeneous, commonly presenting as orbital inflammatory pseudotumor (OIP), scleritis, keratitis, or retinitis.^[2]^ Among these, OIP, also termed idiopathic orbital inflammation or idiopathic orbital inflammatory syndrome, is particularly prevalent and is defined as a benign, noninfectious, inflammatory disorder of unknown etiology that involves various orbital structures.^[3,4]^ In adult orbital disorders, OIP ranks third in incidence following Graves’ ophthalmopathy and lymphoproliferative diseases classification of OIP can be based on the clinical course (acute, subacute, or chronic), histopathological characteristics (classic, granulomatous, sclerosing, or nonspecific), and the anatomical sites involved, such as the lacrimal gland, sclera, extraocular muscles (myositis), optic nerve, diffuse orbital involvement, or orbital apex.^[3,5–10]^ Recognition of these various subtypes assists in accurate diagnosis, prognostication, and selection of appropriate therapeutic strategies. Here, we report a rare case of ANCA-associated sclerosing orbital inflammatory pseudotumor that was refractory to cyclophosphamide and dependent on high-dose corticosteroids, successfully managed with rituximab (RTX). The aim of presenting this case is to raise awareness of ocular-limited AAV as a diagnostic consideration in steroid-dependent orbital disease, and to highlight RTX as a potential steroid-sparing therapeutic option in such refractory cases.

## 2. Case presentation

A 72-year-old Chinese man presented with a 6-month history of recurrent headaches prior to admission. Laboratory tests at another institution revealed positive p-ANCA and c-ANCA, markedly elevated anti-myeloperoxidase (MPO) antibodies (>3836 AU/mL; reference <20 AU/mL), and elevated anti-proteinase 3 (PR3) antibodies (39.6 AU/mL; reference <20 AU/mL). Brain MRI at that time was unremarkable. Based on these findings, a diagnosis of AAV was made, and treatment with oral prednisone acetate (1 mg/kg/d, gradually tapered) plus oral cyclophosphamide (CTX, 50 mg/d) was initiated. The headache improved initially but recurred following corticosteroid tapering.

**Figure 1.**
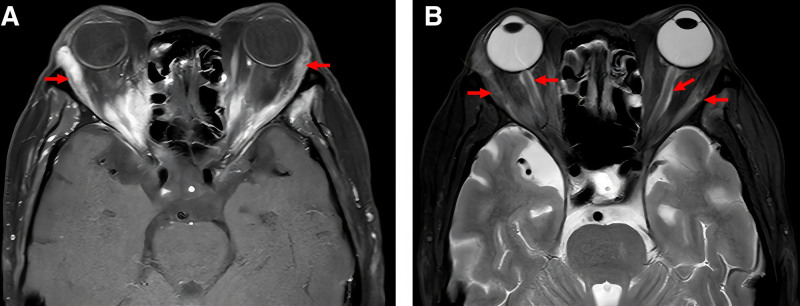
Plain + contrast-enhanced orbital MRI. (A) Bilateral lacrimal gland enlargement with obvious uniform enhancement. (B) Slight bilateral extraocular muscle enlargement with obvious margin enhancement, linear enhancement observed in the bilateral optic nerve sheath regions.

**Figure 2.**
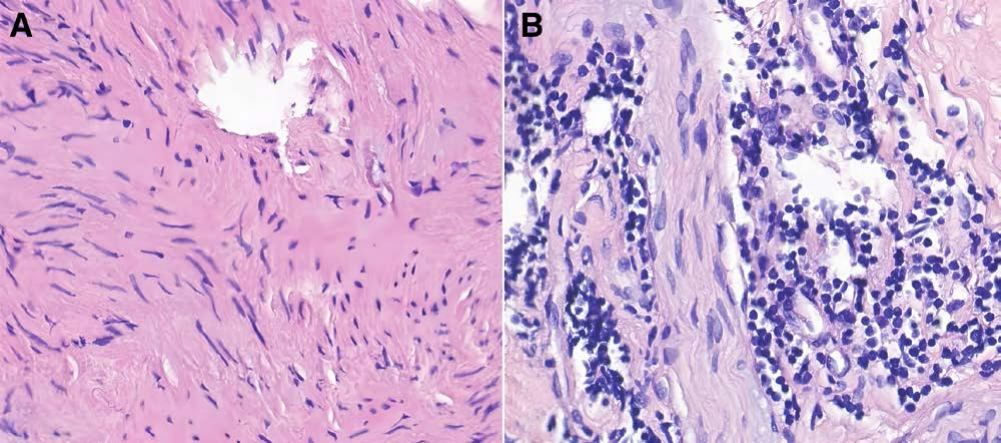
Biopsy of right orbital lesions. (A) Perivascular fibrous tissue proliferation and hyaline degeneration (hematoxylin and eosin staining, 200× magnification). (B) Lymphocytic infiltration (hematoxylin and eosin staining, 200× magnification).

**Figure 3.**
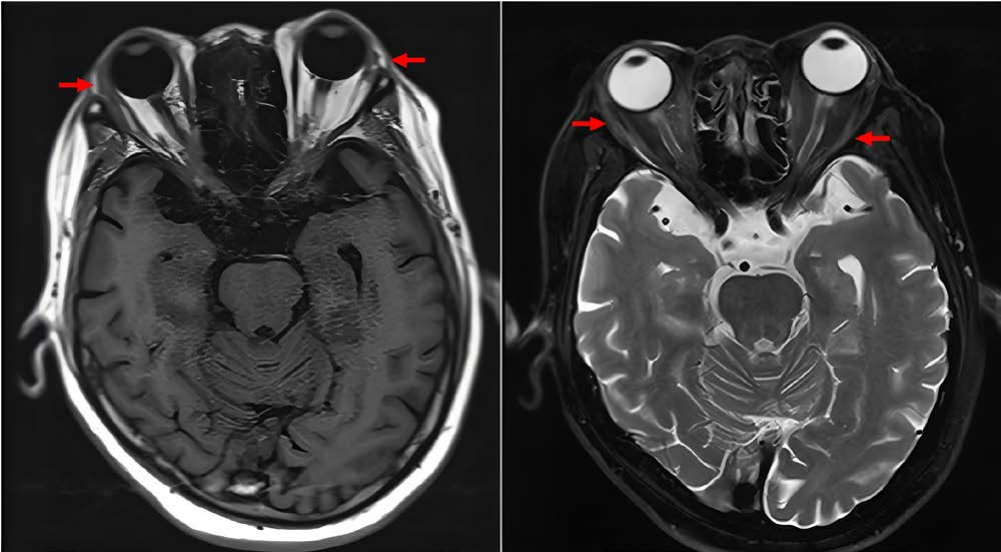
Plain + contrast-enhanced orbital MRI: size reduction was observed in certain lesions.

**Figure 4.**
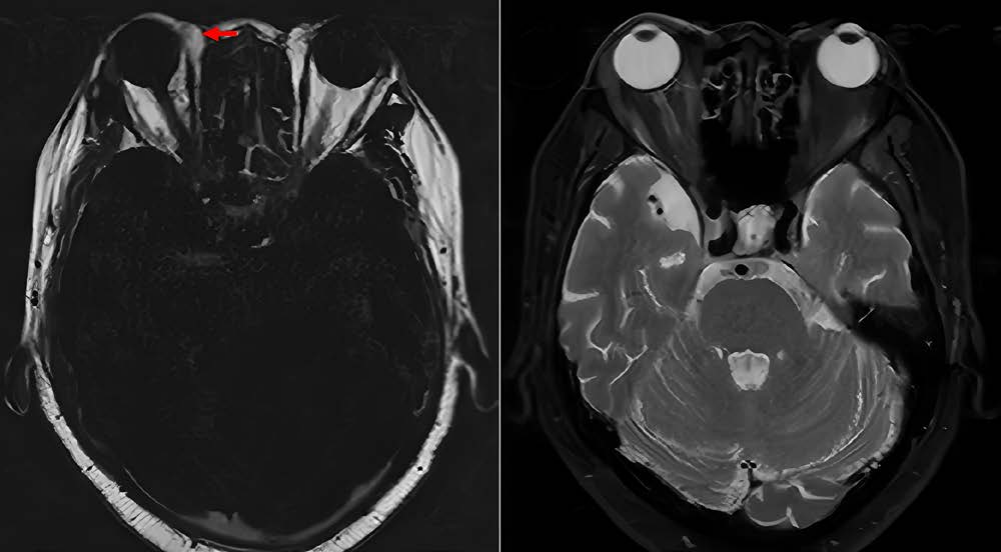
Plain + contrast-enhanced orbital MRI: slight increase in lesions in the space outside the medial and lateral rectus muscles of the right orbit.

After 6 months of therapy, MPO and PR3 antibody titers normalized, but the patient developed worsening headaches, bilateral exophthalmos, and progressive visual loss, prompting referral to our hospital. His medical history included type 2 diabetes mellitus, managed with insulin and metformin. On April 2, 2024, the patient was admitted to the Department of Rheumatology and Immunology at West China Hospital. On admission, inflammatory markers were elevated (C-reactive protein [CRP] 93.70 mg/L; erythrocyte sedimentation rate [ESR] 95 mm/h). LDH was within normal range (153 U/L). Autoimmune and infectious screenings were negative. Patch-like areas of hyperintensity on T2-weighted imaging with evident enhancement on contrast scans were observed in the left temporal and infratemporal fossae, consistent with OIP (Fig. 1). Positron emission tomography–computed tomography ruled out neoplastic lesions and indicated inflammatory changes. Orbital biopsy of the right lesion revealed perivascular fibrosis, hyaline degeneration, and mild lymphocytic infiltration (Fig. 2). During initial treatment with high-dose steroids and CTX, the patient showed clinical improvement. Orbital MRI reexamination suggested shrinkage of the lesion sites (Fig. 3). Treatment with CTX was continued as planned (0.8 g/4 weeks, total 2.4 g). However, headache recurred as the steroid dose was tapered, and repeat orbital MRI indicated enlargement of the orbital lesions (Fig. 4). High-dose intravenous steroids combined with oral CTX (50 mg/d) resulted in initial clinical and radiological improvement. However, relapse occurred during steroid tapering, and symptoms improved again upon dose escalation. Following relapse of the orbital inflammatory pseudotumor during steroid tapering, RTX was administered at 500 mg weekly for 4 consecutive weeks. Substantial resolution of orbital pain, proptosis, and visual symptoms was observed within 8 weeks, enabling complete corticosteroid discontinuation. ANCA titers remained negative throughout RTX therapy and follow-up. No recurrence occurred over the 12-month period, with sustained remission confirmed through serial clinical evaluations conducted approximately every 1–3 months by rheumatology and ophthalmology specialists. Assessments included stable best-corrected visual acuity (Snellen charts, maintained or improved to baseline without deterioration), absence of proptosis on Hertel exophthalmometry (consistently <20 mm bilaterally with no rebound), full and pain-free extraocular motility (target-pursuit testing to exclude diplopia or restrictions), and no recurrence of periorbital swelling or tenderness (palpation and patient-reported symptoms). Posttreatment orbital MRI was not performed due to the patient’s stable clinical status. The detailed clinical course and antibody titers are presented in Table 1.

**Table 1:** ANCA titers and treatment milestones.

Date	MPO–ANCA	PR3–ANCA	Clinical context/comment
2024-03	<1 (reference range <1)	<1 (reference range <1)	First outpatient test at West China Hospital before admission
2024-04-02	<1 (reference range <1)	<1 (reference range <1)	First inpatient test after admission
2024-04-22	12.9 (reference range <20 RU/mL)	<2 (reference range <20 RU/mL)	Before starting glucocorticoids plus cyclophosphamide (post-admission)
2024-07-27	6.38 (reference range <20 RU/mL)	<2 (reference range <20 RU/mL)	Immediately before RTX

Early assays reported results with a cutoff of “<1”; later assays reported RU/mL with a reference range of <20 RU/mL.

ANCA = anti-neutrophil cytoplasmic antibody, MPO = myeloperoxidase, PR3 = proteinase 3, RTX = rituximab.

## 3. Methods

### 3.1. Patient admission and clinical evaluation

The patient was admitted to the Department of Rheumatology and Immunology at West China Hospital on April 2, 2024. A detailed medical history and physical examination were performed, focusing on ocular symptoms, systemic vasculitis manifestations, and comorbidities. Baseline laboratory work-up included complete blood count, liver and renal function tests, CRP, ESR, lactate dehydrogenase (LDH), and autoimmune serology (p-ANCA, c-ANCA, anti-MPO, anti-PR3, ANA, extractable nuclear antigen panel, IgG4, rheumatoid factor, and antinuclear antibodies). Infectious screening included hepatitis panel, tuberculosis IFN-γ release assay, and syphilis testing.

### 3.2. Imaging

Orbital magnetic resonance imaging (MRI) was performed using a 3.0T scanner, including T1-weighted, T2-weighted, and contrast-enhanced sequences. Chest computed tomography (CT) was obtained to assess pulmonary involvement. Positron emission tomography–computed tomography was conducted to exclude neoplasia and assess systemic inflammation. Temporal artery Doppler ultrasonography was performed to exclude giant cell arteritis.

### 3.3. Biopsy and histopathology

An incisional biopsy of the right orbital lesion was obtained under local anesthesia. Tissue samples were fixed in 10% neutral-buffered formalin, embedded in paraffin, sectioned, and stained with hematoxylin–eosin (H&E). Histopathological evaluation focused on the presence of fibrosis, inflammatory cell infiltration, vascular changes, and granuloma formation.

### 3.4. Treatment protocol

The initial treatment regimen consisted of high-dose intravenous methylprednisolone pulses (500 mg/d for 3 days), followed by oral prednisone acetate (1 mg/kg/d, tapered over time) combined with oral CTX (50 mg/d). Upon relapse during steroid tapering, dexamethasone (15 mg/d) was temporarily administered. RTX was subsequently initiated at a dose of 500 mg weekly for 4 consecutive weeks.

### 3.5. Follow-up

The patient was followed for 12 months after RTX initiation, with clinical assessments, orbital MRI, and laboratory evaluations (CRP, ESR, and ANCA titers) performed every 3 months to monitor disease activity and treatment response.

## 4. Discussion

Our patient presented with ANCA-associated inflammatory pseudotumor that relapsed during steroid tapering despite initial remission with glucocorticoid–cyclophosphamide therapy, highlighting steroid dependence and the need for an effective steroid-sparing strategy in the context of comorbid type 2 diabetes mellitus.

RTX is a chimeric monoclonal antibody targeting the CD20 antigen expressed on pre-B and mature B lymphocytes, leading to B-cell depletion via complement-dependent cytotoxicity, antibody-dependent cellular cytotoxicity, and induction of apoptosis. Originally developed for B-cell lymphomas, RTX has since been successfully applied in various autoimmune diseases, including autoimmune hemolytic anemia,^[11]^ where elimination of autoreactive B cells reduces pathogenic antibody production. In oncology, RTX is an established component of treatment regimens for B-cell lymphomas, where its therapeutic effect relies on the elimination of malignant CD20^+^ cells, in part through direct induction of apoptosis.^[12]^ The RAVE trial indicated that the RTX-based regimen was more efficacious than the CTX-based regimen in inducing the remission of recurrent disease in AAV patients, with 67% and 42% of patients of the RTX and control groups respectively reaching the primary end point (*P* = .01).^[13]^ Many other studies have also demonstrated the effectiveness of RTX in the treatment of recurrent or refractory OIP.^[14–19]^ In autoimmune diseases such as ANCA-associated vasculitis, RTX depletes CD20^+^ B cells, including memory B cells and plasmablast precursors, thereby reducing pathogenic autoantibody production, modulating antigen presentation, and suppressing proinflammatory cytokine release. This leads to reduced activation of neutrophils and diminished tissue injury. This highlights RTX’s role as an effective steroid-sparing agent in steroid-dependent or CTX-refractory cases of ANCA-associated ocular disease.

In our patient, RTX was initiated after relapse despite ANCA negativity, at a dose of 500 mg weekly for 4 consecutive weeks. Marked orbital symptom resolution was observed within 8 weeks, enabling complete steroid withdrawal. ANCA remained negative during and after RTX therapy, and no relapse occurred over the 12-month follow-up period. RTX is a chimeric monoclonal antibody targeting the CD20 antigen expressed on pre-B and mature B lymphocytes. By binding CD20, RTX induces B-cell depletion via multiple mechanisms, including complement-dependent cytotoxicity, antibody-dependent cellular cytotoxicity, and direct induction of apoptosis.^[20]^ In autoimmune diseases such as autoimmune hemolytic anemia, RTX has been shown to reduce pathogenic autoantibody production by eliminating autoreactive B-cell clones.^[21]^ In the context of AAV, RTX not only reduces circulating ANCA levels by depleting short-lived plasmablast precursors, but may also suppress ANCA-independent local inflammatory cascades within affected tissues.^[13,22]^ This dual mechanism likely explains the clinical improvement in our patient despite persistent ANCA negativity prior to RTX initiation, suggesting that orbital disease activity may be driven by tissue-resident B cells and local immune mechanisms rather than solely by measurable circulating ANCA. The uniqueness of this case lies in the combination of sclerosing orbital localization, relapse despite ANCA negativity, and the need for steroid-sparing therapy due to comorbid diabetes mellitus. These factors underscore RTX as a rational and effective option in localized, treatment-refractory ocular AAV.

Although anti-retinal or anti-ocular antibodies were not assessed in this patient, such antibodies have been implicated in other autoimmune ocular disorders and could theoretically contribute to tissue-specific inflammation in ANCA-associated disease.^[23,24]^ In addition to humoral mechanisms, ocular pathology in AAV may also be mediated by T cells and innate immune cells infiltrating the orbital tissue, where B cells act as antigen-presenting cells and potent activators of T cell-driven inflammation.^[24,25]^ RTX-mediated depletion of CD20^+^ B cells may therefore confer therapeutic benefit not only through suppression of autoantibody production, but also by disrupting local B–T cell interactions, attenuating proinflammatory cytokine release, and reducing recruitment of innate effector cells. This broader immunomodulatory effect may explain the patient’s marked clinical improvement despite persistently negative ANCA titers.^[26,27]^

ANCAs are marker antibodies of small vessel vasculitis. This includes granulomatosis with polyangiitis, microscopic polyangiitis, and eosinophilic granulomatosis with polyangiitis, which are collectively known as AAV.^[28]^ ANCA-associated ocular disease can occur in individuals of any age who do not suffer concomitant systemic diseases and may present as the sole manifestation.^[2]^ Although the eye is considered an immune-privileged site, vasculitis can affect the sclera, episcleral vessels and limbal vessels, and the retinal and choroidal vessels, thereby causing damage in the natural barriers of the eye and the occurrence of inflammation. ANCA-associated ocular disease most commonly affects the orbit and is usually induced by pseudotumors in granulomatous inflammation. Granulomas may develop from retro-orbital fat but more commonly originate through adjacent structures, particularly those of the lacrimal glands, meninges, and sinuses.^[29]^ Pseudotumors can induce exophthalmos, eyelid swelling, diplopia, orbital pain, lacrimal gland involvement, and optic nerve compression. They are also accompanied by vision loss or even blindness and may ultimately result in enucleation. Besides OIP, other common manifestations include scleritis, episcleritis, and keratitis.^[2,30]^ Inflammatory pseudotumors may occur in up to 30% of granulomatosis with polyangiitis cases, but are rare in eosinophilic granulomatosis with polyangiitis cases and even less commonly reported in microscopic polyangiitis cases.^[31–34]^

In addition to conventional inflammatory markers such as CRP and ESR, other biochemical parameters may provide further insight into disease processes. LDH is a nonspecific yet clinically valuable biomarker that catalyzes the reversible conversion of lactate and pyruvate during glycolysis and gluconeogenesis, and it is ubiquitously expressed across nearly all body tissues. Elevated serum LDH levels indicate cellular membrane disruption and the subsequent release of cytosolic enzymes into the bloodstream, arising from diverse pathological conditions such as acute and chronic inflammation, hemolysis, tissue ischemia, and neoplastic proliferation. In autoimmune disorders like systemic lupus erythematosus, persistently elevated LDH has been linked to increased disease activity and incorporated into assessments of cumulative organ damage risk.^[1]^ In this case, the patient’s LDH level remained within normal limits (153 U/L; reference range: 135–225 U/L), implying a lack of significant systemic tissue damage, hemolysis, or tumor-associated metabolic alterations at the time of assessment, even amid pronounced ocular inflammation. This observation suggests that the inflammatory process was predominantly confined to the orbital region, with minimal systemic extension. Interleukin-6, a multifunctional proinflammatory cytokine pivotal in B-cell differentiation, acute-phase response induction, and autoimmune inflammation regulation, has gained recognition as a promising biomarker and therapeutic target in numerous autoimmune conditions, including vasculitides. Nonetheless, IL-6 was not assayed in this patient, thereby constraining insights into its possible utility for monitoring disease activity here.

This case is notable for several specific features that distinguish it from most previously reported instances of ANCA-associated orbital inflammatory pseudotumor. First, the patient experienced a relapse of orbital disease despite persistent ANCA negativity, yet achieved marked and sustained clinical remission following RTX therapy. This contrasts with the majority of published cases, in which RTX was used predominantly in ANCA-positive or systemically active disease.^[22,29]^ Second, the disease was confined to the orbit without systemic involvement, and the discordance between ANCA titer and clinical activity underscores the possibility that local immune processes—such as tissue-resident B-cell activity—may drive ocular inflammation independently of measurable circulating autoantibody levels. Third, while most literature describes RTX efficacy in systemic AAV or in combination with other immunosuppressants, this case adds novel evidence for its role as a steroid-sparing agent in isolated ocular disease with seronegative relapse. Together, these aspects highlight the rarity of our patient’s presentation and therapeutic course, and suggest that similar patients could benefit from early consideration of RTX even in the absence of ANCA seropositivity.

In summary, a patient with ANCA-associated inflammatory pseudotumor was initially treated with a CTX-based regimen, but experienced recurrence after steroid dose reduction. He was effectively treated after switching to RTX therapy. Further follow-up will be performed to determine the efficacy of the treatment regimen in the longer term.

## 5. Conclusion

This case highlights a rare presentation of ANCA-associated sclerosing orbital inflammatory pseudotumor, initially misresponsive to CTX-based therapy due to steroid dependence. The patient’s condition improved following RTX therapy, aligning with current European Alliance of Associations for Rheumatology recommendations for relapsing AAV. Importantly, disease progression occurred independently of ANCA titers, emphasizing the need for careful clinical monitoring and imaging, particularly when ocular involvement predominates. RTX should be considered as an effective steroid-sparing option in similar refractory or relapsing cases, especially in patients with comorbidities limiting long-term glucocorticoid use.

## Acknowledgments

We thank the patient who made this case report possible.

## Author contributions

**Funding acquisition:** Chaoyang Liang.

**Visualization:** Hua Zhao, Chaoyang Liang.

**Writing – original draft:** Lan Gao, Hua Zhao, Honghu Tang.

**Writing – review & editing:** Lan Gao, Hua Zhao, Honghu Tang.
